# Supplementary material for: Revealing Prognostic and Immunotherapy-Sensitive Characteristics of a Novel Cuproptosis-Related LncRNA Model in Hepatocellular Carcinoma Patients by Genomic Analysis
Source: Cancers (Basel). 2023 Jan 16;15(2):544. doi: 10.3390/cancers15020544 (PMC9857215; doi:10.3390/cancers15020544)
Supplement: Supplementary file 1 [file cancers-15-00544-s001.zip › Table S1.pdf]

Table S1 The clinical characteristics of HCC patients in the training cohort, testing cohort, and total TCGA database.

Characteristics	Levels	Total	Testing cohort	Training cohort	P value
Age, n (%)	<=65	216(62.97%)	87(63.5%)	129(62.62%)	0.9589
	>65	127(37.03%)	50(36.5%)	77(37.38%)	
Gender, n (%)	FEMALE	110(32.07%)	39(28.47%)	71(34.47%)	0.2948
	MALE	233(67.93%)	98(71.53%)	135(65.53%)	
Grade, n (%)	G1	53(15.45%)	19(13.87%)	34(16.5%)	0.8201
	G2	161(46.94%)	69(50.36%)	92(44.66%)	
	G3	112(32.65%)	44(32.12%)	68(33.01%)	
	G4	12(3.5%)	5(3.65%)	7(3.4%)	
	unknow	5(1.46%)	0(0%)	5(2.43%)	
Stage, n (%)	Stage I	161(46.94%)	58(42.34%)	103(50%)	0.3528
	Stage II	77(22.45%)	35(25.55%)	42(20.39%)	
	Stage III	80(23.32%)	37(27.01%)	43(20.87%)	
	Stage IV	3(0.87%)	1(0.73%)	2(0.97%)	
	unknow	22(6.41%)	6(4.38%)	16(7.77%)	
T, n (%)	T1	168(48.98%)	59(43.07%)	109(52.91%)	0.2621
	T2	84(24.49%)	39(28.47%)	45(21.84%)	
	T3	75(21.87%)	34(24.82%)	41(19.9%)	
	T4	13(3.79%)	5(3.65%)	8(3.88%)	
	unknow	3(0.87%)	0(0%)	3(1.46%)	
M, n (%)	M0	245(71.43%)	100(72.99%)	145(70.39%)	1
	M1	3(0.87%)	1(0.73%)	2(0.97%)	
	unknow	95(27.7%)	36(26.28%)	59(28.64%)	
N, n (%)	N0	239(69.68%)	98(71.53%)	141(68.45%)	1
	N1	3(0.87%)	1(0.73%)	2(0.97%)	
