# Supplementary material for: Revealing Prognostic and Immunotherapy-Sensitive Characteristics of a Novel Cuproptosis-Related LncRNA Model in Hepatocellular Carcinoma Patients by Genomic Analysis
Source: Cancers (Basel). 2023 Jan 16;15(2):544. doi: 10.3390/cancers15020544 (PMC9857215; doi:10.3390/cancers15020544)
Supplement: Supplementary file 1 [file cancers-15-00544-s001.zip › Table S2.pdf]

Table S2 Primers used for qRT-PCR

**qRT-PCR primers**	**Sequences (5'-3')**
GAPDH	Forward: TCACCAGGGCTGCTTTTAAC<br>Reverse: GACAAGCTTCCCGTTCTCAG
sn-RNU6	Forward: GTGCTCGCTTCGGCAGCACATATAC<br>Reverse: AAAAATATGGAACGCTTCACGAATTTG
NLRP3	Forward: GATCTTCGCTGCGATCAACAG<br>Reverse: CGTGCATTATCTGAACCCCAC
AL365361.1	Forward: CCAATAAATGGCTCTAAAC<br>Reverse: TAATGGCAGTGACAGGAT
miR-17-5p	Forward: CAAAGTGCTTACAGTGCAGGTAG<br>Reverse: Universal Primer (QIAGEN)
